# Th_17_ responses are not altered by natural exposure to seasonal allergens in pollen-sensitive patients

**DOI:** 10.1186/s13223-016-0157-6

**Published:** 2016-10-24

**Authors:** Agata Schramm, Barbara Jasiewicz-Honkisz, Grzegorz Osmenda, Grzegorz Wilk, Mateusz Siedlinski, Agnieszka Sagan, Pawel T. Matusik, Joanna Maciag, Tomasz Sliwa, Marta Czesnikiewicz-Guzik, Tomasz P. Mikolajczyk

**Affiliations:** 1 Translational Medicine Laboratory, Department of Internal and Agricultural Medicine, School of Medicine, Jagiellonian University, Skarbowa 1, 31-121 Cracow, Poland; 2 Institute of Cardiovascular and Medical Sciences, University of Glasgow, Glasgow, UK; 3 Department of Dental Prophylaxis and Experimental Dentistry, Dental School, Jagiellonian University, Cracow, Poland; 4 Oral Sciences Research Group, Glasgow Dental School, School of Medicine, College of Medical, Veterinary and Life Sciences, University of Glasgow, Glasgow, UK; 5 Institute of Infection, Immunity and Inflammation, University of Glasgow, Glasgow, UK

**Keywords:** Rhinitis, Allergy, Memory cells, Cytokines

## Abstract

**Background:**

Allergic rhinitis affects 10–30 % of the global population and this number is likely to increase in the forthcoming years. Moreover, it commonly co-exists with allergic asthma as a chronic allergic respiratory syndrome. While the involvement of Th_2_ cells in allergy is well understood, alterations of pro-inflammatory Th_17_ responses remain poorly characterized. The aim of our study was to determine whether natural seasonal allergen exposure causes changes in T cell subset characteristics in patients with allergic rhinitis and asthma.

**Methods:**

Sixteen patients with allergic rhinitis/atopic asthma (9M, 7F; age 31.8 ± 12.1) and 16 healthy controls were recruited into the study (9M, 7F; age 31.2 ± 5.3). Blood samples were collected from the patients 1–3 months before pollen season (visit 1), within 7 days of the appearance of pollen/initiation of allergic symptoms (visit 2) and 2 weeks after visit 2 following the introduction of symptomatic treatment with antihistamines (visit 3). Flow cytometry was used to assess major T cell subsets (naïve, central memory, effector memory and CD45RA+ effector) and key T cell cytokine production (IFNγ, IL-17A, TNF and IL-4) using intracellular staining. Data were analyzed using repeated measures ANOVA and paired t test.

**Results:**

As expected, an increase in the percentage of IL‐4+ CD4+ cells was observed during natural pollen exposure in patients with allergic respiratory syndrome. No significant changes were observed in the production of other cytokines, including Th_17_ cells, which tended to be lower than in the control population but unchanged during pollen exposure. Introduction of antihistamine treatment led to only moderate changes in cytokine production from CD4 and CD8 T cells. Selective changes in CD8+ T cells were observed during natural pollen exposure including a decrease in transient cells (with features of CD45RA+ and CD45RO+ cells) and a decrease in the percentage of central memory cells in the peripheral circulation. Within the CD4 cell group the total percentage of CD45RA positive CD4 cells was increased during pollen exposure.

**Conclusions:**

Th_1_ and Th_17_ responses are not altered during pollen season but allergen exposure affects T cell activation and memory cell status in patients with allergic respiratory syndrome.

**Electronic supplementary material:**

The online version of this article (doi:10.1186/s13223-016-0157-6) contains supplementary material, which is available to authorized users.

## Background

Allergic rhinitis affects 10–30 % of the global population [1] and it seems that economic growth and industrialization will only serve to increase this number, not only due to changes in the living environment [2], but also due to expansion of allergic plants caused by increased carbon dioxide concentration in the air [3]. In the Polish population, clinicians diagnose allergic rhinitis in 30.6 % of adults. Among Polish young adults, 16.7 % suffer from seasonal allergic rhinitis [4], which is most frequently caused by allergens such as pollens from trees and grass.

Symptoms of allergic rhinitis and allergic asthma often coexist and it is postulated that they represent a response to the same allergen from upper and lower airways, together named chronic allergic respiratory syndrome [5]. The same inflammatory response is observed in nasal mucosa of patients with allergic rhinitis and bronchial mucosa of patients with asthma. In both of these situations, infiltration by Th_2_ cells is present [5].

Allergen presentation causes differentiation of T cells into Th_2_ cells, which produce cytokines stimulating B cells to produce IgE antibodies. These antibodies bind to mast cells and allergens causing a release of mediators, such as histamine, which are responsible for the symptoms of allergic rhinitis. T cells also take part in the recruitment of eosinophils into the inflammatory site and development of the late phase of allergic reaction [6]. In the allergic reaction Th_17_ may also play a significant role [7]. Although an increased serum level of IL-17 in allergic patients has been described in many studies [8, 9], less is known about changes in Th_17_ cells caused by natural seasonal allergen exposure. Systemic reaction caused by contact with allergen has been widely investigated in many studies, however, the immunological mechanisms leading to primary and secondary responses still remain unclear.

T cells appear to be strongly involved in this process and its subsets may differ depending on antigen stimulation. The CD45RA marker is characteristic for naïve cells, which proliferate when stimulated with antigens, while the presence of CD45RO is characteristic for memory cells, which proliferate during re-call antigen stimulation [10]. Expression of C–C chemokine receptor type 7 (CCR7, CD197) on memory T cells enables distinguishing of central memory T cells (CD45RA− CCR7+), which are able to migrate to the lymph nodes and stimulate dendritic cells, from effector memory T cells (CD45RA− CCR7−), which possess effector functions and can migrate to the inflammatory site [11]. Transcriptional activation of dendritic cells caused by interaction with memory T cells and increased T cell proliferation after culturing with allergen-stimulated dendritic cells in patients suffering from allergic rhinitis has been described by Larsson et al. [12]. After secondary stimulation, central memory T cells lose their CCR7 receptor and acquire effector function becoming effector memory T cells [11].

Although a primary study [11] described the presence of a CD45RA+ effector cell population (CD45RA+ CCR7−) of CD8 cells and lack of this population among a pool of CD4 cells, a newer study revealed the presence of a CD4+ CD45RA+ CCR7− population, which is the most differentiated type [13]. It has been shown that percentages of these cell subsets may differ depending on antigen persistence and load [13].

Allergen-specific naïve and memory T cells differ in allergic and in healthy patients [14]. Changes are also observed depending on whether stimulation is caused by perennial or seasonal allergen. Dust mite-specific cells from mite-allergic patients are mostly of the central memory phenotype, while specific T cells from birch pollen-allergic patients have features of effector memory cells [15].

Several studies have described changes of CD45RO/RA cells in patients with seasonal allergies [16–19], however, less is known about changes in central memory and effector memory cell subsets in these patients. In particular, studies describing the changes caused by pollen exposure in T cell subpopulations of patients suffering from allergic rhinitis are required. Interestingly, expression of surface antigens CCR4, CXCR1 and CD62L on memory cells increases during pollen season only in symptomatic atopic patients, explaining the lack of symptoms in patients with asymptomatic skin sensitization and healthy controls [20].

The aim of our study was to determine whether natural seasonal allergen exposure causes changes in the percentage and immunological status of T cell subsets in patients with allergic respiratory syndrome. To further characterize activation of T cells in this condition, production of cytokines by these cells was assessed.

## Methods

### Study group

Sixteen adult patients with at least a 1 year-long history of pollen allergy manifested by allergic rhinitis or atopic asthma were recruited into the study (Table 1). Allergy was previously confirmed by skin prick tests and by the presence of specific IgE. Patients involved in the study did not present with symptoms at the beginning of the study. If patients suffered from asthma, the disease had to be well controlled prior to participation in the study. At the beginning of the observation, patients were not taking any antihistamines, although they were permitted to use inhaled glucocorticosteroids or inhaled β_2_-mimetics. If a patient had been taking antihistamines prior to the study, treatment was discontinued for at least 2 months before the beginning of observations.

**Table 1:** Clinical characteristics of the patients and a control group

Patient characteristics	Experimental group		Control group	
*Age*	31.8 ± 12.1		31.2 ± 5.3	
*Sex*	M:9; F:7		M:9; F:7	
*Disease*				
Allergic rhinitis	15	(93.8 %)	0	
Asthma	10	(62.5 %)	0	
Allergic conjunctivitis	14	(87.5 %)	0	
Atopic dermatitis	1	(6.25 %)	0	
*Skin prick test positivity*				
Alder	13	(81.3 %)	0	
Hazel	13	(81.3 %)	0	
Birch	12	(75.0 %)	0	
Grass	11	(68.8 %)	0	
*Specific IgE level for skin prick test*—*positive patients [IU/l]*				
Alder	15.3 ± 19.7		N.A.	
Hazel	5.5 ± 7.5		N.A.	
Birch	56.0 ± 39.2		N.A.	
Grass	43.1 ± 46.4		N.A.	
*Specific IgE level for dominant allergen [IU/l]*	41.0 ± 43.7		N.A.	
*Currently smoking*	2	(12.5 %)	1	(6.25 %)
*Treatment*				
Inhaled steroid	7	(43.8 %)	0	
Short acting β_2_-mimetic	2	(12.5 %)	0	
Montelukast	2	(12.5 %)	0	
Long acting β_2_-mimetic	1	(6.25 %)	0	

Age and specific IgE levels are shown as mean ± SD, other features as number of patients

Comorbidity of seasonal allergic rhinitis and asthma was observed in 9 individuals

Coexistence of allergy for tree and grass pollen was observed in 9 patients

Patients with symptoms of perennial allergy or with concomitant allergy to other seasonal allergens with overlapping time of symptom occurrence were excluded from the study. Those suffering from chronic or inflammatory diseases or undergoing immunotherapy within the past 3 years were also excluded.

Blood samples were collected three times from all patients within 1 year: before allergy season (visit 1), during allergy season immediately following the appearance of symptoms (visit 2) and 2 weeks after the appearance of symptoms (visit 3). At the onset of symptoms (visit 2), after blood sample collection, all patients were started on treatment with new antihistamines. Treatment was discontinued after symptoms subsided.

In the period of high pollen concentration, blood samples collected from 16 healthy controls with no history of allergy, with negative skin prick tests for pollens, not taking steroids, antihistamines, β_2_-mimetic nor montelukast, were also examined (Table 1). Investigation protocol was approved by the Jagiellonian University Bioethics Committee, Cracow, Poland (KBET/249/B/2012). Written consent was obtained from all subjects prior to enrollment in the study.

### Blood sample collection

Blood samples were collected from patients into ethylenediaminetetraacetic acid (EDTA) tubes. Whole blood was centrifuged to separate plasma, then peripheral blood mononuclear cells (PBMC) were isolated from the blood by standard gradient centrifugation on Lymphocyte Separation Medium (LSM) 1077 (PAA Laboratories GmbH, Austria).

### Staining protocol

Isolated cells were washed twice with a solution of phosphate-buffered saline (PBS) with 1 % heat inactivated fetal bovine serum (FBS) from Gibco (Life Technologies, USA) and suspended in this buffer. The amount of PBMC was estimated using a Fuchs-Rosenthal chamber. 5 × 10^5^ cells were stained with monoclonal antibodies: anti-CD3–PerCP (Clone SK7), anti-CD4–APC (Clone SK3), anti-CD8–APC-H7 (Clone SK1), anti-CD45 RO–PE (Clone UCHL1), anti-CD45 RA–FITC (Clone L48), anti-CCR7(CD197)–PE-Cy7 (Clone 3D12) from BD Biosciences (San Jose, CA, USA). After staining for 20 min in the dark at 4 °C, cells were washed with PBS+ 1 %FBS and suspended in 200 μl of this buffer. These prepared cells were collected using a FACS Canto II flow cytometer from BD Biosciences (San Jose, CA, USA). Data were analyzed using Flow Jo v10 (Ashland, OR, USA). First, lymphocytes were gated based on forward-scattered and side-scattered light (FSC/SSC). Next, the population of CD3+ cells (T cells) was selected. Among this group, subpopulations of CD4+ and CD8+ T cells were separated (Additional file 1: Fig. S1a). On CD3+, CD4+ and CD8+ cells presence of CD45RA and CD45RO marker was assessed. T cell subpopulations were also analyzed for presence of naïve (CD45RA+ CCR7+), central memory (CD45RA−CCR7+), effector memory (CD45RA−CCR7−) and CD45RA+ effector (CD45RA+ CCR7−) cells.

### Assessment of intracellular cytokines

1 × 10^6^ PBMC were suspended in RPMI 1600 medium (Gibco, Life Technologies, USA) with 10 % FBS, 200 mM l-glutamine and 5 mg/ml gentamicin (Sigma–Aldrich, Saint Louis, MO, USA) and cultured with Leukocyte Activation Cocktail with BD Golgi Plug from BD Biosciences (San Jose, CA, USA) for 4 h at 37 °C in a 5 % CO_2_ humidified atmosphere. After this time, cells were washed with PBS+ 1 %FBS and stained with monoclonal antibodies anti-CD3–PerCP (Clone SK7), anti-CD4–APC (Clone RPA-T4), anti-CD8–APC-H7 (Clone SK1) from BD Biosciences (San Jose, CA, USA). Next, cells were washed with PBS+ 1 %FBS and suspended in Fixation/Permeabilization Solution (BD Biosciences, San Diego, CA, USA). After permeabilization, cells were washed with Perm/Wash/Buffer (BD Biosciences, San Diego, CA, USA) and stained with monoclonal antibodies: anti-IFNγ–FITC (Clone B27), anti-IL-17A–PE (Clone N49-653), anti-TNF–FITC (Clone MAb11) and anti-IL-4–PE (Clone 8D4-8). In some cases, isotype controls: IgG_1_ κ–FITC (Clone MOPC-21) for IFNγ and TNF, IgG_1_κ–PE (Clone MOPC-21) for IL-17A and IL-4 were used (BD Biosciences, San Jose, CA, USA). All staining and permeabilization were performed for 20 min in the dark at 4 °C. After intracellular staining, cells were washed with Perm/Wash Buffer, suspended in PBS+ 1 %FBS and collected using a FACS Canto II flow cytometer from BD Biosciences (San Jose, CA, USA).

### Statistics

The Shapiro–Wilk test was used to test normality of distribution of all analyzed variables. Repeated measures ANOVA was used to test the difference in specific T lymphocyte subpopulations between three visits studied. Subpopulations that significantly differed between three visits were further chosen for paired tests, comparing two visits as well as tests comparing a single visit of study and control groups using t test. All tests were performed in IBM SPSS Statistics (ver. 23). Results are presented as mean ± SEM. *P* values <0.05 were considered as significant.

## Results

### Intracellular cytokine production

**Fig. 1:**
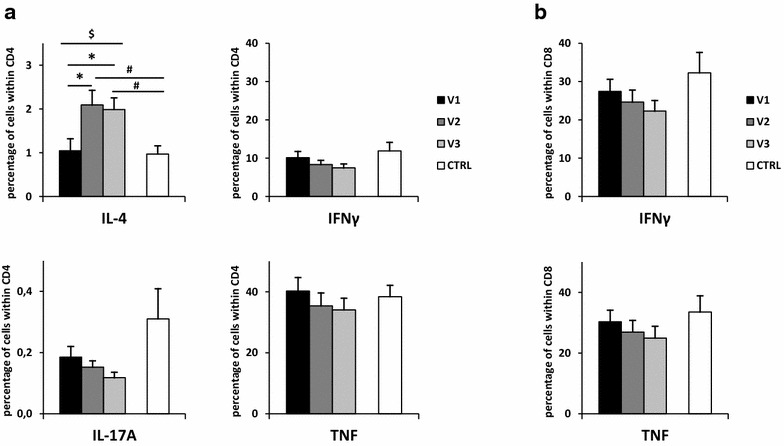
Changes in intracellular cytokine production in T cell subsets during pollen season. Isolated PBMCs were stimulated with leukocyte activation cocktail and were cultured for 4 h. After this time, intracellular production of cytokines was detected in CD4+ T cells (**a**) and in CD8+ T cells (**b**). IL-4 positive or IL-17A positive T cells (**a**) and IFNγ positive or TNF positive T cells (**a** and **b**) are shown. V1—before allergy season, V2—during allergy season immediately following the appearance of symptoms, V3—2 weeks after onset of symptoms, CTRL—control group; data are shown as mean ± SEM; *P < 0.05  in paired samples t test;^ #^ *P* < 0.05 in unpaired samples t test;^ $^ *P* < 0.05 in repeated measures ANOVA, testing all three patients’ visits. Comparisons of cell populations for pairs of single patients’ visits were performed using paired samples t test, while comparisons of cell populations between single patients visit and control subjects were performed using unpaired samples t test

Influence of seasonal allergen exposure on cytokine production was assessed by intracellular staining of IL-4, TNF, IFNγ and IL-17A. A significant increase in IL-4 was observed in CD4+ cells during pollen season (1.0 vs 2.1 vs 2.0 %) (Fig. 1a). There were no changes in the percentages of TNF+ nor IFNγ+ T cells during the visits (Fig. 1a and b). IL-17A was produced by CD4 cells in very small amounts and did not change significantly during pollen season (Fig. 1a).

### CD 45RA/RO

**Fig. 2:**
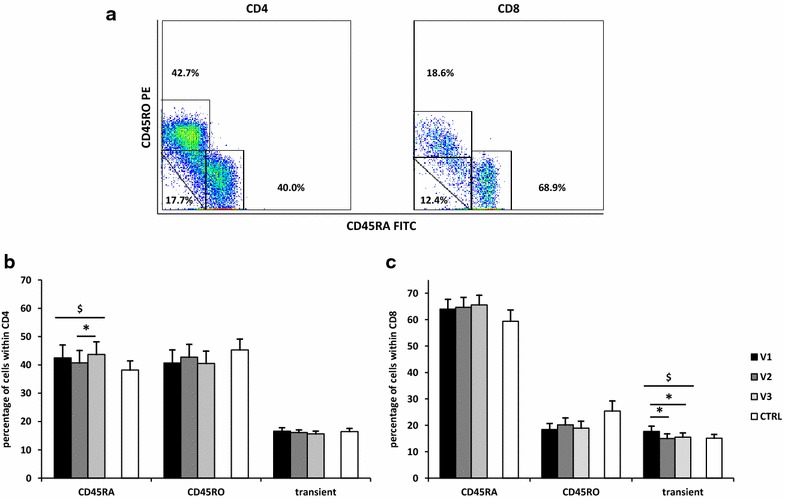
Changes in CD45RA and CD45RO subpopulations of T cell subsets during pollen season. Distribution of CD45RA and CD45RO marker among CD4+ cells (**a** and **b**) and CD8+ cells (**a** and **c**) was analyzed. Cytometric example 2 weeks after the onset of symptoms is shown in (**a**). V1—before allergy season, V2—during allergy season immediately following the appearance of symptoms, V3—2 weeks after the onset of symptoms, CTRL—control group; data are shown as mean ± SEM; *P < 0.05 in paired samples t test;^ $^ *P* < 0.05 in repeated measures ANOVA, testing all three patients’ visits. Comparisons of cell populations for pairs of single patients’ visits were performed using paired samples t test, while comparisons of cell populations between single patients visit and control subjects were performed using unpaired samples t test

Next, to ascertain activation of the immune system caused by seasonal allergens, expression of CD45RA and CD45RO markers in CD4+ and CD8+ cells was investigated (Fig. 2a). The percentage of CD4+ CD45RA positive cells increased in allergic patients during pollen season from 40.8 % at the beginning of pollen season to 43.7 % 2 weeks after onset of symptoms (*P* = 0.03) (Fig. 2b). Among CD8 cells, a decrease in transient cells during pollen season was observed at the beginning of pollen season (15.0 %) and 2 weeks from the onset of symptoms (15.5 %) in comparison to before pollen season (17.7 %) (V1–V2 *P* = 0.01; V1–V3 *P* = 0.03) (Fig. 1c). In all three groups, an increase in the percentage of CD45RO cells at the beginning of pollen season with a decrease 2 weeks later was observed, although only in CD3 cells this trend reached statistical significance (V1—32.8 % vs V2—35.1 % vs V3—32.1 %; *P* respectively 0.01 and 0.004) (data not shown). There were no differences in the percentage of CD45RA and CD45RO cells between allergic patents and the control group.

### Memory Cell Subsets

**Fig. 3:**
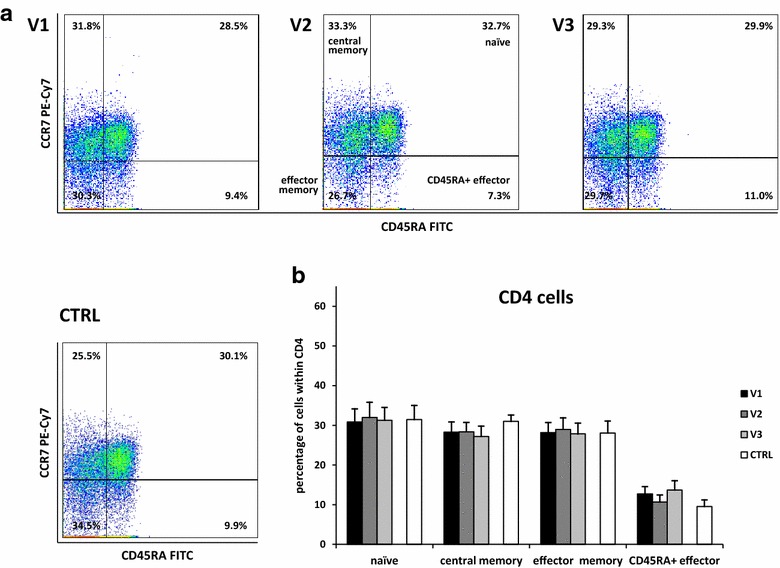
Changes in CD4 memory cell subsets during pollen season. Among CD4 cells the following memory cell subsets were separated: naïve (CD45RA+ CCR7+), central memory (CD45RA− CCR7+), effector memory (CD45 RA− CCR7−), CD45RA+ effector (CD45RA+ CCR7−). Cytometric example (**a**) and changes in the percentages of the subsets (**b**) are shown. V1—before allergy season, V2—during allergy season immediately following the appearance of symptoms, V3—2 weeks after onset of symptoms, CTRL—control group; data are shown as mean ± SEM

**Fig. 4:**
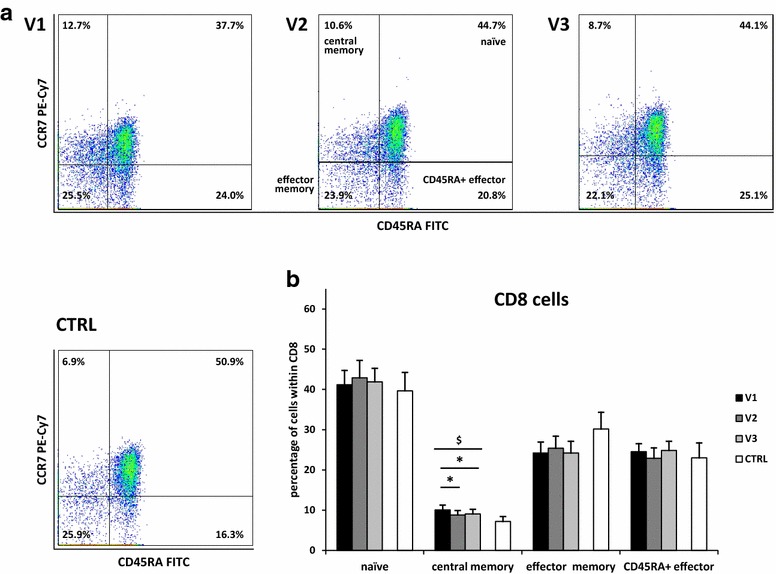
Changes in CD8 memory cell subsets during pollen season. Among CD8 cells the following memory cell subsets were separated: naïve (CD45RA+ CCR7+), central memory (CD45RA− CCR7+), effector memory (CD45 RA− CCR7−), CD45RA+ effector (CD45RA+ CCR7−). Cytometric example (**a**) and changes in percentages of the subsets (**b**) are shown. V1—before allergy season, V2—during allergy season immediately following the appearance of symptoms, V3—2 weeks after onset of symptoms, CTRL—control group; data are shown as mean ± SEM; *P < 0.05 in paired samples t test;^ $^ *P* < 0.05 in repeated measures ANOVA, testing all three patients’ visits. Comparisons of cell populations for pairs of single patients’ visits were performed using paired samples t test, while comparisons of cell populations between single patients visit and control subjects were performed using unpaired samples t test

To investigate the influence of allergen exposure on memory cell subsets we determined percentages of naïve, central memory, effector memory and CD45RA+ effector cells in the group of CD4 and CD8 cells. Allergen exposure did not cause statistically significant changes in the group of CD4 memory cells in pollen-sensitive patients, although an increase in the percentage of CD45RA+ effector cells 2 weeks after the onset of symptoms was close to statistical significance (ANOVA *P* = 0.06) (Fig. 3a and b). In the group of CD8 cells, a decrease in the percentage of central memory cells was observed during allergen exposure in comparison to the state before pollen season (10.1 vs 8.8 vs 9.1 % *P* = 0.01) (Fig. 4a and b).

### T cell subsets

We also determined whether allergen exposure could influence the percentage of T cells. There were no statistically significant differences observed among percentages of CD4+ and CD8+ neither between visits nor in comparison with the control group (Additional file 1: Fig. S1b). The CD4/CD8 ratio also remained constant in allergic patients before and in the allergy season and did not differ from the control group.

## Discussion

With regard to the increasing number of people suffering from allergies, understanding the mechanisms leading to the complex manifestation of symptoms is important in the search for effective treatment methods. As expected, coexistence of seasonal allergic rhinitis and asthma was observed in more than half of the individuals. Determination of the role of T cells and their subsets in seasonal allergy may be crucial to understanding the mechanism of this reaction.

Natural allergen exposure caused a significant increase in IL-4 production by CD4 cells in pollen-sensitive patients. Our findings confirm the previous observation by Wosinska-Becler et al. describing an increase of IL-4 during the peak of birch season in comparison with non-pollen season in birch-sensitive patients [21]. In this group of patients a decrease in IFNγ production in comparison with alder season was observed, but no significant changes were present when compared with non-pollen season, which is also consistent with our results. A study performed in asthmatic children revealed that the percentage of CD4+ cells producing IL-4 and cells producing IFNγ is higher during acute exacerbations than at convalescence [22]. Another study in children with atopic asthma showed an increase in the percentage of CD4 and CD8 cells producing IL-4 and a decrease in CD4 cells producing IFNγ in comparison with healthy controls, while the percentage of cells producing TNF remained unchanged [23]. In addition, the percentage of CD8 cells producing IL-4 spontaneously was higher in children with asthma in this study. Two other studies comparing patients with asthma and healthy controls did not demonstrate any changes in the percentage of cells producing IL-4. One of these studies reported a lower percentage of CD4 cells producing IFNγ in these patients [24], while the second study observed a higher percentage of CD4 and CD8 cells producing IFNγ in atopic asthmatic patients [25]. Bias of results showing changes in patients suffering from allergic asthma can be related to the various kinds of allergens that they were sensitive to (i.e. house dust, animals, moulds), while in our study exposition to seasonal allergen was investigated.

Allergen provocation outside of pollen season in patients with asthma resulted in a decrease in IL-4+ CD4+ cells. In this study, changes in the number of IFNγ-producing CD4 cells depended on response type. A decrease in IFNγ-producing CD4 cells was observed in patients who presented only with early reaction to the allergen challenge, without late-phase reaction [26].

Some studies report an increased percentage of Th_17_ cells in patients with allergic rhinitis. However, in most of these studies, patients allergic to perennial allergens [27, 28] or sensitive to seasonal and perennial allergens were involved [29]. Another study describes the influence of perennial allergen challenge by nebulization on Th_17_ cells [30], however, less is known about changes over time caused by natural seasonal allergen exposure. It was shown that the percentage of IL-17A-producing CD4+ T cells may also depend on polysensitization of the patients and on patient’s sex [29]. Although an increased serum IL-17 level was observed in patients sensitive to perennial allergens, pollen allergens did not cause significant changes [29].

In our study, we did not observe significant changes in the intracellular production of this cytokine. Our results are in opposition to Ciprandi et al. who describe a higher percentage of Th_17_ cells in pollen-sensitive allergic rhinitis patients than in the healthy controls, however, the control group in this study was very small and comparison to the state before pollen season was not performed [31].

Our findings demonstrate the influence of pollen season on memory cells in patients suffering from allergic respiratory syndrome. We did not observe any changes in CD4+ nor CD8+ T cells caused by natural allergen exposure. Our results are consistent with those of Kalrsson et al. who have shown an increase in CD4+ cells in patients suffering from allergic rhinitis during provocation but not during natural antigen exposure [32]. After in vitro stimulation with *P. pratense,* an increased number of circulating allergen-specific CD4+ T cells was seen. In addition, proliferation of CD4+ cells and in some cases, CD8+ cells was observed in grass pollen allergic patients [33]. In pollen-sensitive patients receptor density also changes throughout the year. In these individuals, Monteseirin et al. observed a decrease in the number of CD4+ receptors per cell in the spring [34].

Exposure to seasonal allergens affected expression of CD45 RA/RO isoforms in pollen-sensitive patients. We observed an increase in CD4+ CD45RA+ cells 2 weeks from the beginning of allergy season in comparison with the onset of symptoms. These changes were accompanied by a decrease in transient CD8 cells during pollen season. Although presence of the CD45RA marker is characteristic for naïve cells and CD45RO maker for memory cells, re-call antigen stimulated change of CD4 cells from RA+ to RO+ phenotype is a gradual process during which two transitional subsets co-expressing RA and RO are present: CD45RA^bright^/RO^bright^, which are present in very small amount, and CD45RA^dull^/RO^dull^ expressing both isoforms at low level, possessing mixed features of CD45RA+ and CD45RO+ cells. Moreover, CD45RO+ cells under certain conditions can re-acquire the RA marker [35]. Cells co-expressing CD45RA and CD45RO have been described in patients with atopic asthma, and these subsets form 31–46 % of the T helper cells [36]. In our study transient cells formed on average, depending on visit, 15.7–16.6 % of CD4 cells and 15.0–17.7 % of CD8 cells in allergen-sensitive patients and did not differ from the control group. In children with allergic asthma an increase in the absolute number of CD8+ T cells expressing CD45RO is associated with a decrease in percentage, but not in the absolute number of CD4+ CD45RO positive cells when compared with healthy controls [37]. We did not observe any differences in the percentage of CD4 and CD8 RO positive cells between allergic patients and the control group. Although we have seen an increase in CD3 cells expressing CD45RO marker at the beginning of allergen season with a decrease 2 weeks later, these changes were not significant either on CD4 or CD8 cells. Similar findings, representing transformation of lymphocytes from naïve into memory type cells caused by contact with allergen, were described previously by Pawlik et al. on CD4 cells, who however, observed an increase in CD4+ 45RO+ lymphocytes in the blood of patients with seasonal symptoms during allergy season [38]. A decrease in the percentage of CD3+ CD45RO+ cells two weeks after the onset of symptoms in our study can be explained by migration of CD45RO+ cells into the inflammatory site [39]. The mechanisms of this process are complex and may include various factors such as oxidative stress, which lead to inflammation [40].

Investigation of skin biopsies obtained from pollen-allergic patients challenged with intradermal injection of allergen demonstrated increased frequency of memory cells 24 h after challenge in comparison with stimulation with control solution, suggesting a role for these cells in the cutaneous late-phase reaction [41]. Immunocytochemical analysis of nasal mucosa of patients suffering from seasonal allergic rhinitis revealed an increase in CD4+ CD45RO+ cells during pollen season [42]. The presence of activated memory T cells in mucosa was also demonstrated using flow cytometry, however, there were no differences between allergic and non-allergic patients. Moreover, these findings were present only at the inflammatory site and did not correlate with peripheral blood mononuclear cells [43].

A recent study demonstrated an increased number of effector/memory T cells (CD4+CD25low) in patients with seasonal allergic rhinitis 6 h after nasal allergen challenge with grass pollen. This study described changes in peripheral blood cells, although allergen challenge was induced only once and may not have accurately reflected the changes caused by natural exposure during allergy season [44]. In patients with allergic rhinitis, the response to provocational exposure may be different from the response during pollen season [32].

We observed a decrease in CD8 central memory cells during pollen season. These changes reflect the differentiation of memory cells in the allergic reaction, with CD45RA+ effector cells being the most differentiated type. A non-significant increase in CD45RA+ effector cell subpopulation of CD4 cells appears 2 weeks after the first presentation of symptoms (visit 3) when compared with the measurement performed at the onset of symptoms (visit 2). This is probably due to the time needed to convert effector memory cells into CD45RA+ effector cells. In this period of time all patients were receiving treatment with new antihistamines, which were shown to be highly effective in relieving symptoms of seasonal allergic rhinitis [45, 46]. This medication was not administered during the previous visit (at the onset of symptoms) and could have also affected our results. Although the influence of H_2_-blockers on CD4+ RO+ cells and suppressor cells was previously demonstrated [19], less is known about the influence of new H_1_-blockers on these cells. However, the effect of the first generation H_1_-blocker, clemastine, on natural killer and antibody-dependent cellular cytotoxicity activities of lymphocytes have been described [47].

## Conclusions

In summary, our data shows that natural seasonal allergen exposure in patients with allergic respiratory syndrome affects T cell activation and their memory status, without altering Th_1_ and Th_17_ responses. Additional studies are required to better understand the complexity of cellular reactions in patients with allergic respiratory syndrome.

## Additional file

**Additional file 1: Fig. 1.** T cell populations in allergic patients and in the control group. Lymphocytes were gated based on forward-scattered and side-scattered light (FSC/SSC). Next, the population of CD3+ cells was selected. Among this group, subpopulations of CD4+ and CD8+ cells were separated (**a**). Next, changes in the T cell populations in allergic patients during pollen season and comparison with the control group were determined (**b**). V1—before allergy season, V2—during allergy season immediately following the appearance of symptoms, V3—2 weeks after onset of symptoms, CTRL—control group; data are shown as mean±SEM.

## Abbreviations

CCR: C–C chemokine receptor
CD: cluster of differentiation
EDTA: ethylenediaminetetraacetic acid
FBS: fetal bovine serum
FSC: forward-scattered
IFNγ: interferon γ
IgE: immunoglobulin E
IL: interleukin
LSM: lymphocyte separation medium
PBMC: peripheral blood mononuclear cells
PBS: phosphate-buffered saline
SSC: side-scattered
Th: T helper
TNF: tumor necrosis factor
