# Supplementary figures and images for: Th17 responses are not altered by natural exposure to seasonal allergens in pollen-sensitive patients
Source: Allergy Asthma Clin Immunol. 2016 Oct 24;12:55. doi: 10.1186/s13223-016-0157-6 (PMC5078933; doi:10.1186/s13223-016-0157-6)

**A**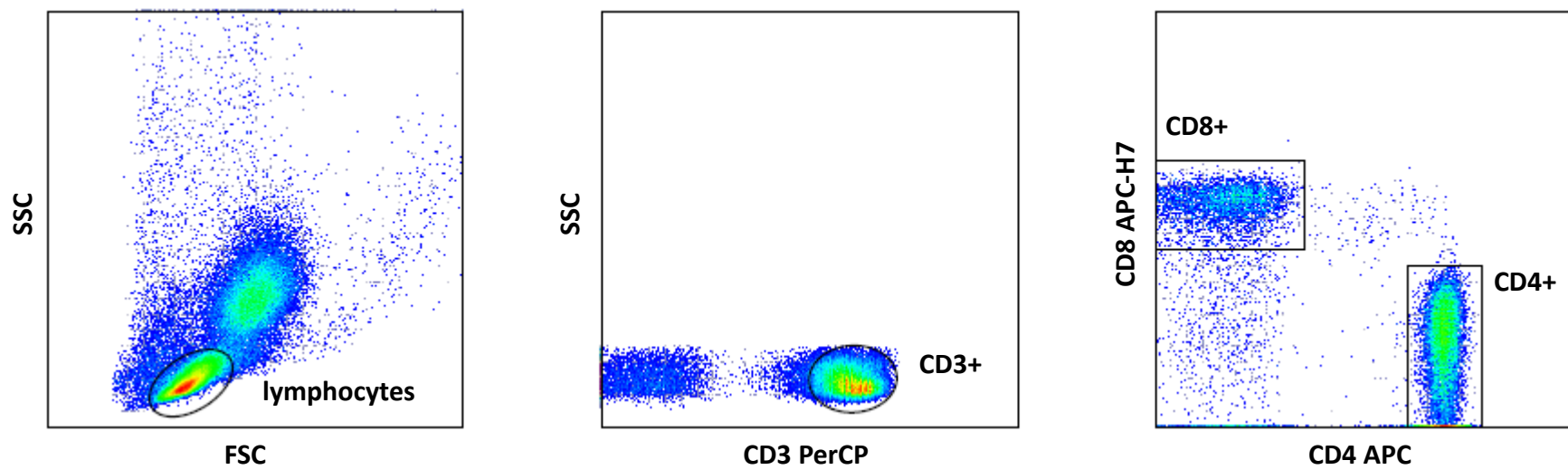**B**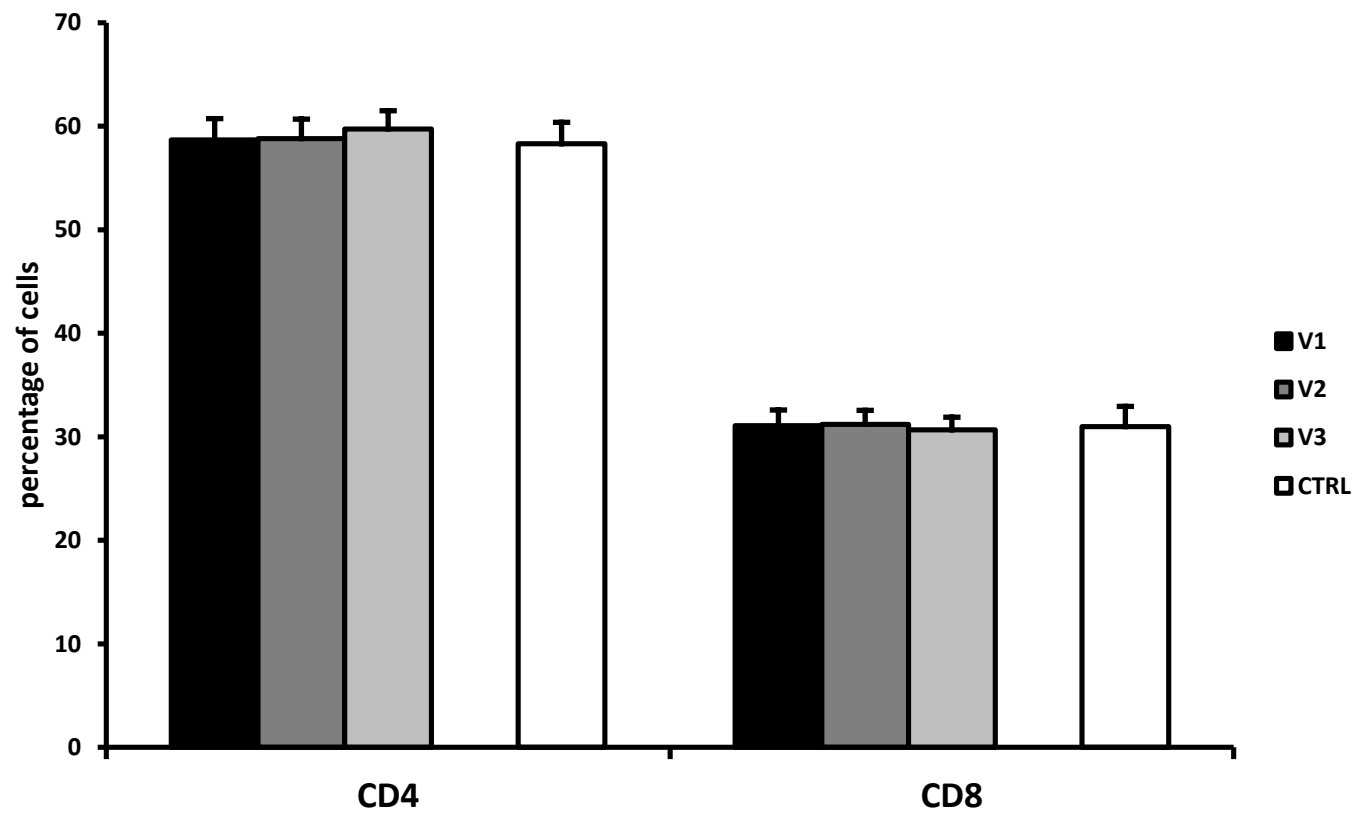**Supplementary Figure 1.**

Supplement: Supplementary file 1 — Additional file 1: Fig. 1. T cell populations in allergic patients and in the control group. Lymphocytes were gated based on forward-scattered and side-scattered light (FSC/SSC). Next, the population of CD3+ cells was selected. Among this group, subpopulations of CD4+ and CD8+ cells were separated (a). Next, changes in the T cell populations in allergic patients during pollen season and comparison with the control group were determined (b). V1—before allergy season, V2—during allergy season immediately following the appearance of symptoms, V3—2 weeks after onset of symptoms, CTRL—control group; data are shown as mean±SEM. [file 13223_2016_157_MOESM1_ESM.pdf]
